# Bickerstaff’s Brainstem Encephalitis and Miller Fisher Syndrome: A Rare Overlap

**DOI:** 10.7759/cureus.55000

**Published:** 2024-02-26

**Authors:** Nikhil Pantbalekundri, Sourya Acharya, Samarth Shukla, Sunil Kumar, Suprit Malali

**Affiliations:** 1 Department of Medicine, Jawaharlal Nehru Medical College, Datta Meghe Institute of Higher Education and Research, Wardha, IND; 2 Department of Pathology, Jawaharlal Nehru Medical College, Datta Meghe Institute of Higher Education and Research, Wardha, IND

**Keywords:** guillain-barré syndrome, bilateral facial nerve palsy, miller fisher syndrome (mfs), gbs variant, bickerstaff's brainstem encephalitis

## Abstract

A rare illness known as "Bickerstaff's brainstem encephalitis" (BBE) is characterized by an abrupt brainstem dysfunction and includes the triad of diminished consciousness, ataxia, and ophthalmoplegia. It differs from the Guillain-Barré syndrome (GBS) and Miller Fisher syndrome (MFS) by involving the central nervous system (CNS) and frequently manifesting as reduced consciousness. Here, we describe a rare instance of Bickerstaff's encephalitis coexisting with MFS, where the patient had rapidly progressing quadriplegia, VII cranial nerve palsy, and episodes of unconsciousness.

## Introduction

Progressive bilateral ophthalmoplegia, ataxia, and/or pyramidal symptoms are the hallmarks of Bickerstaff's brainstem encephalitis (BBE) [1]. According to a Japanese survey, there are 100 new cases of BBE every year, and the yearly incidence is 0.078/100 000 [2]. Although the etiology of the illness is still not entirely known, it is linked to an autoimmune process that is brought on by an earlier infection. The characteristic symptoms of disorientation or sleepiness have been mentioned in the majority of case reports of BBE. While the Miller Fisher variant, Bickerstaff's encephalopathy, and Guillain-Barré syndrome (GBS) are closely knit, overlap presentations are likely. Following is a unique occurrence for a patient with BBE who also had progressive quadriplegia and VII cranial nerve palsy, a possible Fisher-Bickerstaff variant.

## Case presentation

A 24-year-old male presented to the emergency department in an unresponsive state with a prior history of weakness in both limbs for the past 20 days. He had no prior medical history. The patient was intubated due to a poor Glasgow Coma Scale (GCS) when he arrived at the emergency department. The patient’s kin described progressively weakening of both legs to the point that nine days following the onset of symptoms, the patient found it difficult to get out of bed. It was gradual in onset and gradually progressive. He then experienced two episodes of vomiting and lost consciousness, which is when he was taken to the emergency room. The older brother denied having any symptoms of photophobia, neck restrictions, or headaches.

On physical examination, the patient had a fever of 38°C. Blood pressure was 140/90 mm Hg, heart rate was 115/min, and oxygen saturation was 98% in volume control mode with a fraction of inspired oxygen (FiO2) of 0.5. A neurological examination was done when the patient was conscious and followed commands. It revealed diminished tone, absent power, and areflexia. Cranial nerve examination revealed a bilaterally sluggish pupillary response to light. There were features of bilateral infranuclear facial nerve palsy. His plantar reflexes were silent bilaterally. He had episodes of drowsiness throughout the day, and his GCS score oscillated between 3/15 and 10/15. Regular blood tests revealed that the C-reactive protein (CRP) was 2.3 mg/L and the white blood cells (WBC) were 9900/cm. The rest of the blood investigations were within normal limits.

A screen for antinuclear antibodies came out negative. The cerebrospinal fluid (CSF) examination revealed albumin-cytological dissociation, a normal leucocyte count, and normal glucose levels. A computed tomography (CT) of the thorax, abdomen, and pelvis, which was performed for the possibility of paraneoplastic encephalomyelitis, was reported as normal. A head MRI revealed nothing noteworthy. Motor axonal polyneuropathy was discovered via a nerve conduction investigation. Cytomegalovirus, enterovirus, varicella-zoster virus, human herpesvirus, and herpes (deoxyribonucleic acid) DNA viral indicators were all negative. Antiganglioside antibody serology also came up negative.

At first, Miller Fisher syndrome (MFS) and viral encephalitis were considered. However, a diagnosis of encephalitis is improbable because of a cellular CSF and a negative viral screen. Further consideration was given to a broad differential, which included autoimmune processes, botulism, cerebral neoplastic and paraneoplastic encephalomyelitis, and more. Investigations revealed a negative autoimmune screen, negative antibodies, and a normal MRI brain. BBE is frequently accompanied by anti-GQ1b antibodies or antiganglioside antibodies. Although our patient tested negative for anti-GQ1b, only 70% of BBE patients had serum GQ1b immunoglobulin (IgG) antibodies that were positive [3]. A diagnosis of "overlap of BBE and MFS" was made. The patient was started on intravenous IgGs at a dose of 2 g/kg divided over five days from day 3 of admission. The patient was closely monitored for consciousness, respiration, and neurological improvement. Neuro-physiotherapy was intensified. Tracheostomy was done on day 8 of intubation. With improved consciousness, respiratory drive, and ventilation, the patient was gradually weaned off of the mechanical ventilator. The power in all limbs gradually improved, and on examination, the patient had a power of 3/5 in all limbs and could hold his neck up for 30 seconds by day 18 of his intensive care stay. Tracheostomy care and limb physiotherapy were continued throughout the stay. With improved general condition, the patient was mobilized with the help of walkers and gradually with sticks. The patient was discharged with a power of 3/5 in all four limbs. Patient lost follow-up after discharge.

## Discussion

BBE was referred to as "a grave syndrome with benign prognosis" by Edwin Bickerstaff in the 1950s [4]. He described a condition that included ataxia, sleepiness, and ophthalmoplegia and was preceded by infection [5]. There have been comparisons drawn between GBS and MFS, including areflexia and an elevated protein level in the CSF. 

Bickerstaff distinguished BBE from MFS by noting that only BBE exhibits disordered consciousness. A study was conducted by Odaka et al. [6] to describe the neurological characteristics of 62 BBE cases. Profound sensory impairment (16%), hyperreflexia (34%), Babinski's sign (40%), and disturbed consciousness (drowsiness 45%; stupor, semicoma, or coma 29%) were all central nervous system (CNS) signs. Other typical neurological symptoms were nystagmus, bulbar palsy, and facial paralysis. Additionally, there have been case reports of BBE patients exhibiting hypersomnolence and decorticate posture, which indicate CNS involvement [7-9]. Research has illuminated potential underlying pathways, despite the fact that the pathophysiology of BBE is still not completely understood. Based on the effects of sera on the blood-brain barrier, Saito et al. [10] conducted a study to explain the phenotypic variations between MFS and BBE. It has now been proposed that the three illnesses are components of a continuous clinical spectrum that affects both the CNS and the peripheral nervous system. Notably, serum GQ1b IgG antibody positivity in BBE cases is reported to be 70%; not all cases of BBE are anti-GQ1b positive. Comparatively, GQ1b antibodies are present in 83-100% of MFS patients [11-13] and 8% of GBS patients [14]. According to research by Liu et al., the majority of neuromuscular junctions (NMJ) in extraocular muscles as well as nerve terminals inside muscle spindles were bound by an anti-GQ1b antibody. On the other hand, there were few anti-GQ1b ganglioside antibodies binding to NMJs in the leg and axial muscles [15]. Recent findings in favor of anti-GQ1b antibody-positive BBE versus anti-GQ1b antibody-negative BBE were published by Yoshikawa et al. [16].

Antiganglioside antibodies are thought to function by molecularly mimicking infectious organisms in a number of BBE cases, according to research [17, 18]. They include *Salmonella typhi*, *Mycoplasma pneumoniae*, *Campylobacter jejuni* infection, Epstein-Barr virus, varicella-zoster virus, coronavirus, measles virus, and herpes simplex virus. Koga released a paper in 2013 that examined the clinical features of BBE and was based on a national survey conducted in Japan [1]. Typical BBE was linked to a good prognosis and recovery, but atypical BBE was distinguished by a delayed recovery and aberrant CSF and brain MRI findings. He came to the conclusion that BBE was comprised of both typical and atypical cases. The diagnosis of the BBE and MFS overlap (Fisher-Bickerstaff overlap syndrome) is made using a mix of anamnestic, clinical, and radiological findings because there is no particular biological marker for the condition [19]. BBE does not currently have a proven treatment. Intravenous immunoglobulin and plasmapheresis are the standard treatments, although additional clinical trials are needed to determine their efficacy. Although this is constrained by the rarity of the condition, further research is required to understand the processes of BBE, define its relationship to MFS and GBS, and assess the efficacy of treatment.

## Conclusions

Patients with ataxia, ophthalmoplegia, and CNS involvement who can manifest as altered consciousness, autonomic instability, pseudobulbar affect, decreased consciousness, and/or pyramidal signs should be suspected of having BBE or overlap syndromes.

MFS, GBS, and BBE belong to a spectrum of illnesses with an underlying autoimmune mechanism that is frequently brought on by an earlier infection and still needs to be better understood. Intravenous immunoglobulins and electrophoresis are used as treatments, and the majority of patients recover well.

## References

[1] Koga M (2013). A nationwide survey of patients with Bickerstaff brainstem encephalitis: diversity of underlying mechanism (Article in Japanese). Rinsho Shinkeigaku.

[2] Koga M (2013). Bickerstaff brainstem encephalitis: epidemiology, diagnosis, and therapy (Article in Japanese). Nihon rinsho.

[3] Odaka M, Yuki N, Yamada M, Koga M, Takemi T, Hirata K, Kuwabara S (2003). Bickerstaff's brainstem encephalitis: clinical features of 62 cases and a subgroup associated with Guillain-Barré syndrome. Brain.

[4] BI ER (1957). Brain-stem encephalitis: further observations on a grave syndrome with benign prognosis. Br Med J.

[5] Bickerstaff ER, Cloake PC (1951). Mesencephalitis and rhombencephalitis. Br Med J.

[6] Odaka M, Yuki N, Hirata K (2001). Anti-GQ1b IgG antibody syndrome: clinical and immunological range. J Neurol Neurosurg Psychiatry.

[7] Wakerley BR, Soon D, Chan YC, Yuki N (2013). Atypical Bickerstaff brainstem encephalitis: ataxic hypersomnolence without ophthalmoplegia. J Neurol Neurosurg Psychiatry.

[8] Shimozono K, Shimono K, Kusunoki S (2012). Case of Bickerstaff brainstem encephalitis associated with spindle coma and decorticate posture (Article in Japanese). Rinsho Shinkeigaku.

[9] Acharya S, Andhale A, Shukla S (2021). Hyperacute Gullain Barre Syndrome (GBS); the catastrophic Variant- a rare case report. J Pharm Res Int.

[10] Saito K, Shimizu F, Koga M (2013). Blood-brain barrier destruction determines Fisher/Bickerstaff clinical phenotypes: an in vitro study. J Neurol Neurosurg Psychiatry.

[11] Ito M, Kuwabara S, Odaka M, Misawa S, Koga M, Hirata K, Yuki N (2008). Bickerstaff's brainstem encephalitis and Fisher syndrome form a continuous spectrum: clinical analysis of 581 cases. J Neurol.

[12] Chiba A, Kusunoki S, Obata H, Machinami R, Kanazawa I (1993). Serum anti-GQ1b IgG antibody is associated with ophthalmoplegia in Miller Fisher syndrome and Guillain-Barré syndrome: clinical and immunohistochemical studies. Neurology.

[13] Willison HJ, Veitch J (1994). Immunoglobulin subclass distribution and binding characteristics of anti-GQ1b antibodies in Miller fisher syndrome. J Neuroimmunol.

[14] Kim JK, Bae JS, Kim DS (2014). Prevalence of anti-ganglioside antibodies and their clinical correlates with Guillain-Barré syndrome in Korea: a nationwide multicenter study. J Clin Neurol.

[15] Liu JX, Willison HJ, Pedrosa-Domellöf F (2009). Immunolocalization of GQ1b and related gangliosides in human extraocular neuromuscular junctions and muscle spindles. Invest Ophthalmol Vis Sci.

[16] Yoshikawa K, Kuwahara M, Morikawa M, Kusunoki S (2020). Bickerstaff brainstem encephalitis with or without anti-GQ1b antibody. Neurol Neuroimmunol Neuroinflamm.

[17] Lahole S, Acharya S, Raisinghani N (2021). Para infectious Guillain-Barre syndrome [GBS) in covid-19: a case report. J Evol Med Dent Sci.

[18] Roos RP, Soliven B, Goldenberg F, Badruddin A, Baron JM (2008). An elderly patient with Bickerstaff brainstem encephalitis and transient episodes of brainstem dysfunction. Arch Neurol.

[19] Abide Z, Sif Nasr K, Kaddouri S, Edderai M, Elfenni J, Salaheddine T (2023). Bickerstaff brainstem encephalitis: a case report. Radiol Case Rep.

